# Modelling average maximum daily temperature using *r* largest order statistics: An application to South African data

**DOI:** 10.4102/jamba.v10i1.467

**Published:** 2018-05-02

**Authors:** Murendeni M. Nemukula, Caston Sigauke

**Affiliations:** 1 Department of Statistics and Operations Research, University of Limpopo, South Africa; 2 Department of Statistics, University of Venda, South Africa

## Abstract

Natural hazards (events that may cause actual disasters) are established in the literature as major causes of various massive and destructive problems worldwide. The occurrences of earthquakes, floods and heat waves affect millions of people through several impacts. These include cases of hospitalisation, loss of lives and economic challenges. The focus of this study was on the risk reduction of the disasters that occur because of extremely high temperatures and heat waves. Modelling average maximum daily temperature (AMDT) guards against the disaster risk and may also help countries towards preparing for extreme heat. This study discusses the use of the *r* largest order statistics approach of extreme value theory towards modelling AMDT over the period of 11 years, that is, 2000–2010. A generalised extreme value distribution for *r* largest order statistics is fitted to the annual maxima. This is performed in an effort to study the behaviour of the *r* largest order statistics. The method of maximum likelihood is used in estimating the target parameters and the frequency of occurrences of the hottest days is assessed. The study presents a case study of South Africa in which the data for the non-winter season (September–April of each year) are used. The meteorological data used are the AMDT that are collected by the South African Weather Service and provided by Eskom. The estimation of the shape parameter reveals evidence of a Weibull class as an appropriate distribution for modelling AMDT in South Africa. The extreme quantiles for specified return periods are estimated using the quantile function and the best model is chosen through the use of the deviance statistic with the support of the graphical diagnostic tools. The Entropy Difference Test (EDT) is used as a specification test for diagnosing the fit of the models to the data.

## Introduction

Most of the classical statistical techniques that are frequently used in the energy sector and meteorological analysis are classified into regression analysis, time series, state space and Kalman filtering (Hahn, Meyer-Nieberg & Pickl 2009). The limitation that is commonly encountered among such techniques is that they concentrate on the mean instead of the tails of the distributions. This leads to unreliable estimates as most of the sample values fall outside the tails of the distribution, as well as the difficulty in estimating the model parameters that would lead to a good fit in the tails (Byström 2005; Gencay & Selcuk 2004; Sigauke et al. 2012; Soares & Scotto 2004). The problems that arise as consequences of using statistical techniques that do not concentrate on the tails of the distributions are overcome by the use of extreme value theory (EVT) because of its ability to model the asymptotic behaviour of thin- or heavy-tailed distributions (Gencay & Selcuk 2004). According to Hyndman and Fan (2010), the frequency of the occurrence of coldest or hottest temperatures is an extreme event and is best modelled with the use of EVT. Most of the scientific areas including actuary, energy forecasting and meteorology are associated with the thin- or heavy-tailed data and hence consider the use of EVT techniques (Gencay & Selcuk 2004). Zhang et al. (2014) used the change point approach of EVT in modelling stationary annual flood peaks during 1951–2010 in the Pearl River basin, China.

In this study, the use of the *r* largest order statistics approach in modelling average maximum daily temperature (AMDT) in South Africa is discussed. This approach is an extension of the block maxima approach. The primary purpose is to present a modelling approach that is suitable for modelling the frequency of the occurrence of extremely high temperatures and heat waves as one of the natural hazards. The meteorological data that are used comprise the daily average maximum temperatures that are collected by the South African Weather Service over the period 2000−2010. The daily average maximum temperatures are specified in this study by defining the non-winter season as the period from September to April of each year. The problem to be addressed is the demonstration of the modelling approach that contributes towards disaster risk reduction as far as the occurrence of extreme high temperatures is concerned. The R package that is used for modelling the *r* largest order statistics in this article is the ismev package (an introduction to statistical modelling of extreme values) that is developed by Heffernan and Stephenson (2016).

The *r* largest order statistics (for *r* > 1) is fitted to the average maximum temperature. This is performed in an effort to assess the asymptotic behaviour of the *r* largest order statistics within the blocks of equal lengths. The use of the generalised extreme value (GEV) approach in modelling temperature gains attention worldwide. Wang et al. (2013) used the GEV inferential analysis in modelling historical changes in Australian temperature extremes. The use of ordinary GEV distribution for block maxima (*r* = 1) is criticised in the literature in favour of the *r* largest order statistics model (*r* > 1). Bader, Yan and Zhang (2015) argue that the *r* largest order statistics approach is widely used in extreme value analysis because it may use more information from the data than just the block maxima. ‘Modelling only block maxima is a wasteful approach to extreme value analysis if other data on extremes are available’ (Coles 2001:74). The major limitation of extreme value analysis is the scarcity of the extremes and, consequently, the classifications of extreme value modelling that are more detailed than just fitting an ordinary GEV distribution are essential (Chikobvu & Sigauke 2013; Coles 2001). Because of this limitation, the use of the GEV distribution for *r* largest order statistics (GEV_r_) gains preference over the ordinary GEV distribution for block maxima. Coles (2001) and Soares and Scotto (2004) argue that it is not usual to get data that are suitable to be modelled using the GEV distribution for *r* largest order statistics, and also that the GEV distribution for *r* largest order statistics becomes wasteful of the data if one block contains more extremes than other blocks. The advantage of the peaks-over-threshold (POT) as an alternative EVT approach is that it avoids blocking and hence uses as much as possible the available data. However, the motivation for studying the block maxima approach in this study is based on Ferreira and De Haan (2015), where it is argued that the block maxima approach is a more efficient method under usual practical conditions, but has not been studied thoroughly in comparison to the POT approach.

The rest of the article is organised as follows: A brief discussion of disaster risk management and reduction is given in the section ‘Disaster risk management and reduction’. The ‘Models’ section presents the extreme value analysis techniques that are used in this study. The empirical results are presented and discussed in the ‘Empirical results and discussion’ section. The ‘Conclusion and recommendation’ section concludes and recommends the possible areas for further research. Appendix 1 is provided at the end.

## Disaster risk management and reduction

Twigg (2015) views disasters as short- or long-term extreme sudden events that may result from various factors or hazards including floods, earthquakes and heat waves. The occurrences of natural disasters contribute towards major global problems that include, among others, health issues and loss of lives. The global economy also faces enormous repercussions in the presence of disasters based on the fact that the supply chain may be disturbed once a disaster occurs in some particular countries. Some of the incidents of disasters are the earthquake that occurred in Japan and floods that were experienced in Thailand, both during 2011. Such incidents result in disastrous impacts that might affect the supply chain worldwide. This is because of the fact that Japan and Thailand are the industrial suppliers of certain goods and services (Twigg 2015). Though it is impractical to combat the prevalence of natural disasters, the usual practice is to try as much as possible to reduce the risk thereof. There are several procedures with which the risk of natural disasters is reduced, depending on the type of disaster under consideration. This study focuses on the reduction and management of the disaster risk that occurs as a result of extreme high temperatures that lead to global change and heat waves.

In the presence of hottest days, people switch on the cooling systems until a point at which all the cooling systems are on, resulting in an extreme increase in electricity demand (Munoz et al. 2010). To this effect, modelling the occurrence of extreme high temperatures is vital in the energy sector. Steffen, Hughes and Perkins (2014) emphasise that heat waves are the contributing factors towards the occurrence of several natural disasters which affect economies and lives of people worldwide. There are several disastrous impacts of heat waves, some of which are highlighted in this study. The presence of extreme heat leads to drought and health consequences, which account for numerous cases of hospitalisations and deaths (Lyon 2009; Meehl & Tebaldi 2004; Noji 2000; Steffen et al. 2014). During the occurrence of heat waves and hot spells, there is a higher demand for water because water reservoirs become low, and also the fact that water may be used for guarding against the risk of fire in various situations (Lyon 2009; Meehl & Tebaldi 2004; Noji et al. 2000; Steffen et al. 2014). The civil constructions such as roads and buildings also turn to be at risk of collapsing because of extreme heat, thereby putting lives of people in danger (Lyon 2009; Meehl & Tebaldi 2004; Noji et al. 2000; Steffen et al. 2014). The agricultural sectors face severe challenges in the presence of extreme heat to an extent that livestock starve and perish because of drought and health challenges that arise as a consequence of extreme heat (Lyon 2009; Meehl & Tebaldi 2004; Noji et al. 2000; Steffen et al. 2014). Accurate and precise statistical modelling of the occurrence of extreme high temperatures is necessary for the purpose of guarding against the risk of the occurrence of such disastrous impacts because of hot spells or heat waves.

## Models

This study is aimed at fitting the GEV distribution for *r* largest order statistics (GEV_r_) on the AMDT. Our objectives include, among others, the use of the maximum likelihood estimation (MLE) method in estimating the target parameters, the choice of the best model out of several nested models using the likelihood ratio test that is based on the deviance statistic, the use of entropy difference test (EDT) in assessing the goodness of fit of the models as well as the estimation of the return levels using the quantile function.

### Generalised extreme value distribution

The GEV distribution is discussed by Fisher and Tippett (1928) as the most suitable approximation for modelling the maxima or minima of a long sequence of finite variables. Suppose that the independent and identically distributed (i.i.d.) finite sequence X_1_,…,X_*n*_ constitutes a random sample of size *n* that is chosen from the random variable X whose marginal distribution function is *F*. Let *M*_*n*_ = max{X_1_,…,X_*n*_}, then the extremal types theorem states that for the suitable normalising constants {*a*_*n*_ > 0} and {*b*_*n*_}, the distribution function of the re-scaled block maxima ${\text{M}}_{n}^{*}$ is given by:

$$Pr\left\{\frac{\left(M_{n}-b_{n}\right)}{a_{n}}\leq x\right\}=F^{n}(a_{n}x+b_{n})\to G(x)\text{ as }n\to \infty$$ [Eqn 1]

for a non-degenerate distribution function *G*, then *G* belongs to a unified GEV distribution family that is given by:

$$G_{\gamma }(x)=\text{exp}\left\{-{\left[1+\gamma \left(\frac{x-\mu }{\sigma }\right)\right]}^{-\frac{1}{\gamma }}\right\}$$ [Eqn 2]

defined for $\left\{x:1+\gamma \left(\frac{x-\mu }{\sigma }\right)>0\right\}$,

where − ∞ < *μ* < ∞ is the location parameter, *σ* > 0 is the scale parameter and − ∞ < *γ* < ∞ is the shape parameter.

The ordinary GEV distribution in equation 2 converges to one of the three families of extreme value distributions, depending on the rate of decay of the tail that is indexed by the shape parameter *γ*. When *γ* = 0, *G_γ_*(*x*) reduces to a type I or a short-tailed unbounded Gumbel family of distributions (Beirlant et al. 2004). When *γ* < 0, *G_γ_*(*x*) is thin-tailed and we get a type II family which is the Weibull class of distributions with an upper bound given by (Beirlant et al. 2004):

$$\mu -\frac{\sigma }{\gamma }.$$

If *γ* > 0, then *G_γ_*(x) belongs to a type III family which is a heavy-tailed Frèchet class of distributions that is bounded below by (Beirlant et al. 2004):

$$\mu -\frac{\sigma }{\gamma }.$$

The survival distribution of the GEV distribution is given by:

$$Pr(X>x)=1-G_{\gamma }(x)=1-\text{exp}\left\{-{\left[1+\gamma \left(\frac{x-\mu }{\sigma }\right)\right]}^{-\frac{1}{\gamma }}\right\}$$ [Eqn 3]

defined for $\left\{x:1+\gamma \left(\frac{x-\mu }{\sigma }\right)>0\right\}$ and *y* = 0.

By letting *p* = *Pr*(*X* > *x*) and rearranging equation 3, we get the quantile function that is given by:

$$x_{p}=\mu +\frac{\sigma }{\gamma }\left\{\left[-\ln{\left(1-p\right)}^{-\gamma }\right]-1\right\},\gamma \neq 0.$$ [Eqn 4]

As *p* → 0 and $\gamma <0,x_{p}=\mu -\frac{\sigma }{\gamma }$.

The quantile function given in equation 4 is used in estimating high quantiles and predicting the probability of exceedance levels in section ‘Empirical results and discussion’. The GEV distribution in equation 2 is then extended to give an asymptotic model for the joint distribution of the *r* largest order statistics within annual blocks, for fixed values of *r*. We initially define:

$$M_{n}^{k}=\text{kth largest of }\{{\text{X}}_{1},\ldots ,{\text{X}}_{n}\}$$ [Eqn 5]

and then identify the limiting behaviour of this variable, for fixed *k*, as *n* → ∞. If equation 1 holds, then, for fixed *k*:

$$Pr\left\{\frac{\left(M_{n}^{\left(k\right)}-b_{n}\right)}{a_{n}}\leq x\right\}\to G_{k}(x)\text{ as }n\to \infty$$ [Eqn 6]

defined on $\left\{x:1+\gamma \left(\frac{x-\mu }{\sigma }\right)>0\right\}$.

where:

$$G_{k}(x)=\text{exp}\left\{-\tau (x)\right\}\sum_{s=0}^{k-1}\frac{\tau {\left(x\right)}^{s}}{s!}$$ [Eqn 7]

with:

$$\tau (x)={\left[1+\gamma \left(\frac{x-\mu }{\sigma }\right)\right]}^{-\frac{1}{\gamma }}.$$ [Eqn 8]

The GEV distribution for the *r* largest order statistics:

$$\begin{array}{l} M_{n}^{\left(r\right)}=(\frac{M_{n}^{\left(1\right)}-b_{n}}{a_{n}},\frac{M_{n}^{\left(2\right)}-b_{n}}{a_{n}},\ldots ,\\ \;\frac{M_{n}^{\left(r-1\right)}-b_{n}}{a_{n}},\frac{M_{n}^{\left(r\right)}-b_{n}}{a_{n}}) \end{array}$$ [Eqn 9]

is the joint probability density function given in Coles (2001) as:

$$\begin{array}{l} f\left(x^{\left(1\right)},\ldots ,x^{\left(r\right)}\right)=\text{exp}\left\{-{\left[1+\gamma \left(\frac{x^{\left(r\right)}-\mu }{\sigma }\right)\right]}^{-\frac{1}{\gamma }}\right\},\\ \;\times \prod_{k=1}^{r}\frac{1}{\sigma }{\left[1+\gamma \left(\frac{x^{\left(k\right)}-\mu }{\sigma }\right)\right]}^{-\frac{1}{\gamma }-1} \end{array}$$ [Eqn 10]

valid for − *∞* < *μ* < *∞, σ* > 0 and − *∞* < *γ* < *∞;* *x*^(*r*)^ ≤ *x*^(*r*–1)^ ≤ …≤ *x*^(1)^; and:

$x^{\left(k\right)}:1+\gamma \left(\frac{x^{\left(k\right)}-\mu }{\sigma }\right)>0$ fir *k* = 1,2,…,*r*.

The likelihood function for the *r* largest order statistics model when *γ* = 0 is given by:

$$\begin{array}{l} L(\mu ,\sigma ,\gamma )=\prod_{i=i}^{m}(\text{exp}\left\{-{\left[1+\gamma \left(\frac{x_{i}^{\left(r_{i}\right)}-\mu }{\sigma }\right)\right]}^{-\frac{1}{\gamma }}\right\}\\ \;\times {\prod_{k=1}^{r_{i}}\frac{1}{\sigma }\left[1+\gamma \left(\frac{x_{i}^{\left(k\right)}-\mu }{\sigma }\right)\right]}^{-\frac{1}{\gamma }-1}) \end{array}$$ [Eqn 11]

defined for $1+\gamma \left(\frac{x_{i}^{\left(k\right)}-\mu }{\sigma }\right)>0,k=1,\ldots ,r_{i},i=1,\ldots m$.

However, the validity of the GEV distribution for *r* largest order statistics depends on the choice of the number of order statistics *r*, which is critical. If *r* is too large, the asymptotic support for the model is likely to be violated, leading to the occurrence of bias (Bader et al. 2015; Coles 2001). Similarly, if *r* is too small, few observations will be generated and the variance of the estimator can be high (Bader et al. 2015; Coles 2001). The details of the asymptotic theory of order statistics that are highlighted in this study are obtained in various parts of the literature, including Falk and Wisheckel (2016).

## Empirical results and discussion

### Modelling using the *r* largest generalised extreme value distribution

A plot of the AMDT in degrees Celsius using data for the non-winter season (September–April of each year) is given in Figure 1 for the period 2000–2010. The results of the *r* largest asymptotic order statistics model that is fitted to the 10 largest annual temperatures over a period of 11 years are summarised in Table 1. Several values of the maximised negative log-likelihood estimate (*λ*_*i*_), for *i* = 1,*…*,10, have led to the maximum likelihood estimates of the target parameters as illustrated in Table 1 with standard errors in parentheses. However, the attention is limited to *r* ≤ 6 order statistics as a result of the reasonable doubt on the validity of the model for *r* ≥ 7. The maximised negative log-likelihood estimates (*λ*_*i*_) seem to be stable for *r* ≤ 6 and start decreasing significantly when *r* ≥ 7. The standard errors of estimates increase significantly when *r* ≥ 7, implying poor fit of the model when *r* ≥ 7. According to Soares and Scotto (2004), the above-mentioned observations also support the doubt of the fitted GEV_r_ distribution when *r* ≥ 7. For *r* ≤ 6, the standard errors of the estimates ($\hat{\mu },\hat{\sigma },\text{ and }\hat{\gamma }$) decrease as the values of *r* increase, implying an increase in precision of the model (Bader et al. 2015; Coles 2001; Soares & Scotto 2004).

**FIGURE 1:**
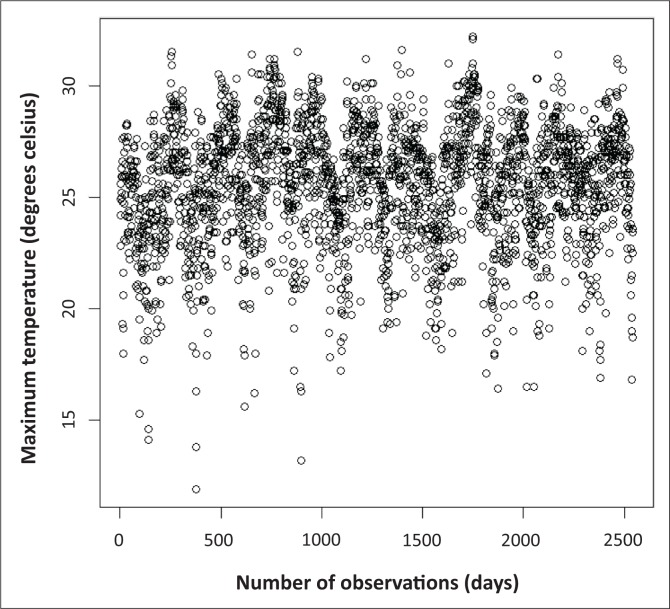
Plot of average maximum daily temperature (°C) for the period 2000−2010. Only data for the period September–April of each year are included.

**TABLE 1:** Maximised log-likelihoods *λ_i_*, parameter estimates $\hat{\mu },\hat{\sigma },\text{ and }\hat{\gamma }$ and standard errors (in parentheses) of *r* largest order statistics model fitted to the temperatures in South Africa with different values of *r*.

*r*	λ_i_	$\hat{\mu }$		$\hat{\sigma }$		$\hat{\gamma }$		95% confidence interval for γ
		Parameter estimate	Standard error	Parameter estimate	Standard error	Parameter estimate	Standard error	
1	−12.5255	30.8813	0.3141	0.9551	0.2643	−0.6848	0.2376	(−1.1505; −0.2191)
2	−15.9670	31.0993	0.2243	0.7540	0.1133	−0.6206	0.1784	(−0.9703; −0.2709)
3	−11.3280	31.1311	0.1660	0.6220	0.0688	−0.4803	0.1173	(−0.7102; −0.2504)
4	−8.5826	31.1190	0.1576	0.6107	0.0616	−0.4515	0.1062	(−0.6597; −0.2433)
5	−0.8328	31.1405	0.1427	0.5743	0.0551	−0.4226	0.0887	(−0.5965; −0.2487)
6	−11.1508	31.1421	0.1358	0.5571	0.0535	−0.3963	0.0824	(−0.5578; −0.2348)

The sufficient evidence of the validity of the Weibull distribution family towards modelling of AMDT in South Africa is revealed by the estimates of the shape parameter for all values of *r*. A negative value of the shape parameter in classical EVT implies that the GEV distribution converges towards the Weibull class of distributions. All estimates of $\hat{\gamma }$ in Table 1 are negative values and the Weibull class of distributions is the most appropriate. The graphical diagnostic tools (probability–probability [P–P] plot and quantile–quantile [Q–Q] plot) for assessing accuracy in the fit of the annual maximum temperature to the *r* largest asymptotic GEV distribution are given for each value of *r* in Figures 2, Table 1-A1 and Table 2-A1. However, the paramount attention in checking the fit of the models in this study is limited to the Q–Q plots based on the fact that the linear pattern of the Q–Q plot is unlikely to be affected by the shifts in the location, scale and symmetry of the distribution, an advantage which is not the case in the P–P plot. Looking at Figures 2 and Table 1-A1, the Q–Q plots for *r* = 2, 3 and 4 display the dots that are near linear. There is doubt on the fit of the model at *r* ≥ 6. The model fitted for *r* = 4 with *λ*_4_ = − 8.5826 seems to be the one that possesses a reasonably good fit. The graphical diagnostics which are the P–P, Q–Q, return level and the density plots are given for *r* = 4 in Figure 3-A1. To justify the appropriateness of the Weibull class of distributions as a proper model for the AMDT in South Africa, the confidence interval for *γ* is estimated for all values of *r*. All the point estimates of the shape parameter *γ* are found to be negative values. It is important to compare the outcomes of point estimation with the results of interval estimation. If indeed the point estimates of the shape parameter are negative values, then they must be bounded below and above by the negative confidence limits. For example, the confidence interval for *γ* considering *r* = 1 is given by:

$$\begin{array}{l} \hat{\gamma }\pm z\alpha /2\times (\text{standard error})\Rightarrow -0.6848\pm 1.96\times 0.2376\\ \;=(-1.1505;-0.2191). \end{array}$$

**FIGURE 2:**
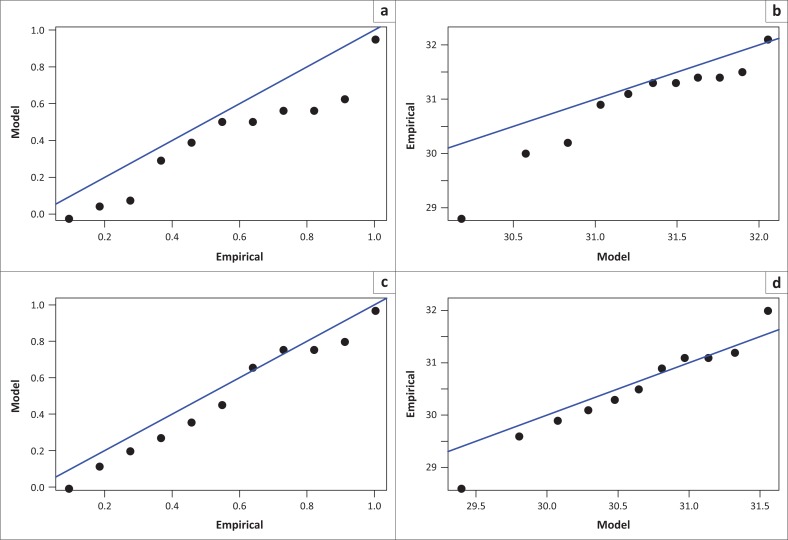
Diagnostic plots illustrating the fit of the data (annual average maximum temperature) to the generalised extreme value distribution for *r* largest order statistics model with *k* = 1 and *k* = 2, (a) probability–probability plot for *k* = 1, (b) quantile–quantile plot for *k* = 1, (c) probability–probability plot for *k* = 2, and (d) quantile–quantile plot for *k* = 2.

This leads us to conclude that at a 95% level of confidence, the value of the shape parameter *γ* is expected to be enclosed within the interval (−1.1505; −0.2191), which makes sense because $\hat{\gamma }=-0.6848$ when *r* = 1. The confidence interval for *γ* is then estimated for all *r* values, and it is noted that all the upper limits are negative values, and hence enclose the point estimates $\hat{\gamma }$. This justifies that the Weibull class of distributions can be used for modelling AMDT in South Africa. These results are included in the last column of Table 1.

There are various tests that are considered in the literature as relevant to assess the goodness of fit of the models to the data. The Anderson–Darling test is suitable for assessing the goodness of fit of the heavy-tailed distributions. In this article, we use the EDT that is discussed in Bader et al. (2015) as a specification test for assessing the goodness of fit of GEV_r_ for *r* largest order statistics (*r* > 1). This is derived as the difference in entropy of GEV_r_ and GEV_r-1_ models. Considering a continuous random variable *X* with density function *f*, the entropy difference is given by:

$$E\left[-\ln f(x)\right]=-\int_{-\infty }^{\infty }f(x)\log f(x)dx,$$ [Eqn 12]

which is the mean of the negative log-likelihood that is estimable by the sample average of the contribution to the log-likelihood from the observed data. Suppose that *r* − 1 top order statistics fit the GEV_r-1_ distribution. Then the difference in the log-likelihood between GEV_r-1_ and GEV_r_ provides a measure of deviation from the null hypothesis $H_{0}^{r}$. However, large deviation from the expected difference under $H_{0}^{r}$ suggests a possible misspecification of the null hypothesis (Bader et al. 2015). From the log-likelihood contribution, the difference in log-likelihood for the *i* th block:

$$X_{ir}(\theta )=l_{i}^{\left(r\right)}-l_{i}^{\left(r-1\right)}$$ [Eqn 13]

is given by:

$$\begin{array}{l} X_{ir}(\theta )=-\log\sigma -(1+\gamma z_{ri}{)}^{-\frac{1}{\gamma }}+(1+\gamma z_{ri-1}{)}^{-\frac{1}{\gamma }}\\ \;-\left(\frac{1}{\gamma }+1\right)\log(1+\gamma z_{ri}),\text{where }z_{i}=\gamma \left(\frac{x_{i}^{\left(r_{i}\right)}-\mu }{\sigma }\right). \end{array}$$ [Eqn 14]

It is critical to test the goodness of fit of the GEV_r_ distribution with a sequence of null hypotheses (Bader et al. 2015). This study desires to test the null hypothesis $H_{0}^{r}$: the GEV_r_ distribution fits the sample of the *r* largest order statistics well for *r* = 1,…,R, where R is the maximum, predetermined number of top order statistics to test. The extreme value analysis (EVA) R package of Bader and Yan (2016) is used for conducting the EDT in this article.

The results of the test are summarised in Table 2, which shows in column 1 the values of order statistics *r* to be tested. The *p-*values from the individual tests at each value of *r* in column 2 establish *r* = 4 as the most appropriate GEV_r_ model to the data. This is based on *p* = 0.7834 which is the highest probability value. Basically, the rule is to reject $H_{0}^{r}$ if *p*-value ≤ *α*, where *α* is the level of significance. At 10% level of significance, we fail to reject $H_{0}^{r}$ when *r* = 4, implying that GEV_r=4_ is the most appropriate model for AMDT in South Africa. Looking at the test statistics in column 4, the critical value *Z*_α/2_ = 1.645 also supports that we fail to reject $H_{0}^{r}$ when *r* = 4. The *p*-values of the Forward and the Strong Stops are also provided together with parameter estimates for each value of *r* in Table 2.

**TABLE 2:** Entropy difference test for diagnosing generalised extreme value distribution for *r* largest order statistics.

*r*	*p*	ForwardStop	StrongStop	Statistic	$\hat{\mu }$	$\hat{\sigma }$	$\hat{\gamma }$
2	0.061593823	0.4240636	0.49817771	1.8692084	31.05364	0.7615547	−0.5923202
3	0.001607416	0.5442273	0.07780948	3.1545576	31.13994	0.6271697	−0.4868675
4	0.783431762	0.8155366	0.10330571	0.2748496	31.10788	0.6074107	−0.4538394
5	0.096269066	0.1012236	0.01989029	1.6632167	31.12734	0.5824586	−0.4292671

### Return level estimation and model selection

Estimates of extreme quantiles and probabilities of exceedance levels of the annual maximum distribution are obtained using the quantile function given in equation 4. These are calculated with the use of parameter estimates of model *r*_4_ which is established as the best fitting model to the data. The quantity *x*_*p*_ in equation 4 is the return level associated with the return period 1/*p*. The level *x*_*p*_ is expected to be exceeded on average once every 1/*p* years (Coles 2001). The deviance statistic is used in assessing the goodness of fit of the models. This is given by:

$$\begin{array}{l} D_{\left(i,j\right)}=2\ln\left(\frac{\lambda (r_{i})}{\lambda (r_{j})}\right)=2\left[\ln\lambda (r_{i})-ln\lambda (r_{j})\right]\sim \chi_{1}^{2},\\ \text{ for }i,j=1,2,\ldots ,6(i\neq j) \end{array}$$ [Eqn 15]

where *λ*(*r*_*i*_) and *λ*(*r*_*j*_) are the maximum likelihood functions of *r*_*i*_ and *r*_*j*_, respectively. A test of the validity of model based on *r*_*i*_ relative to *r*_*j*_ at the *α* level of significance is to reject *r*_*i*_ if *D*_(i,j)_ > *Cα*, where *Cα* is the (1 – *α*) quantile of the $\chi_{1}^{2}$ distribution (Coles 2001; Smith 1989; Soares & Scotto 2004).

**FIGURE 4-A1:**
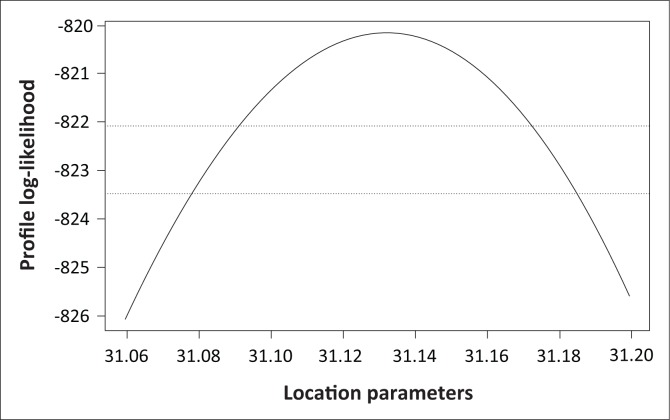
Profile log-likelihood for the location parameter μ using estimates of the model with *r* = 4.

**FIGURE 5-A1:**
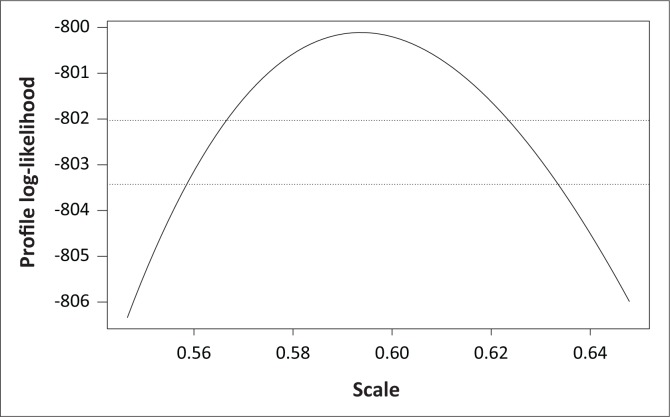
Profile log-likelihood for the scale parameter *σ* using estimates of the model with *r* = 4.

**FIGURE 6-A1:**
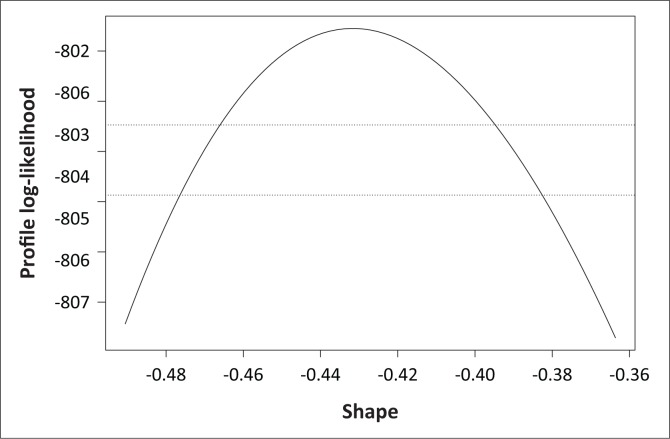
Profile log-likelihood for the shape parameter *γ* using estimates of the model with *r* = 4.

The point estimates ${\hat{x}}_{p}$ for the 5, 10, 25, 50 and 100 years return levels corresponding to the return period 1*/p* are calculated using equation 4 and the outcomes are summarised in Table 3. Looking at 25 years return level, for example, the level *x*_0.04_ = 32.15 is expected to be exceeded on average once every 25 years. For the greater accuracy in the estimation of the return level and parameters in the model for *r* = 4, profile log-likelihoods for *µ, σ* and *γ* are given in Figures 4-A1, 5-A1 and 6-A1, respectively. Table 4 shows the comparison of the models using the deviance statistic given in equation 15. For example, the deviance statistic for comparing *λ*(*r*_1_) and *λ*(*r*_2_) is calculated as *D*_(1,2)_ = 2 (−15.9670 – (−12.5255)) = −6.88. Similar to Soares and Scotto (2004), the deviance statistics in this article are significant when comparing the log-likelihoods among *D*_(2,3)_, *D*_(3,4)_ and *D*_(4,5)_. The comparison is invalid for *D*_(1,2)_ and *D*_(5,6)_. The test for selecting the best model is conducted at 1% level of significance for which $\chi_{1}^{2}=6.64$. For *D*_(2,3)_ and *D*_(4,5)_, *λ*(*r*_3_) and *λ*(*r*_5_) are rejected as *D*_(2,3)_ > 6.64 and *D*_(4,5)_ > 6.64. It is therefore clear that *D*_(3,4)_ = 5.49 < 6.64 reveals failure to reject *λ*(*r*_4_), which implies the validity of *r* = 4 as an order statistic that possesses the reasonably good fit as suggested by the graphical diagnostic tools. The Q–Q and the P–P plots given in Figure 1-A1 show that the Weibull family is the appropriate distribution for the maximum temperatures and also that *r* = 4 possesses the most reasonable fit of the model out of the six order statistics. Equation 4 is also used for estimating the future return levels (extreme quantiles) for the different return periods as illustrated in Table 5. For example, the 90th quantile is calculated as follows:

$$\begin{array}{l} x_{0.1}=31.1190+\frac{0.6107}{0.4515}\left\{1-{\left[-\ln(0.90)\right]}^{0.4515}\right\},\\ \;\Rightarrow x_{0.1}=31.98\approx 32.0. \end{array}$$

**TABLE 3:** Return values for 5, 10, 25, 50 and 100 years.

5 years	10 years	25 years	50 years	100 years
31.78	31.98	32.15	32.24	32.30

**TABLE 4:** Deviance statistics and *p*-values.

D(1,2)	D(2,3)	D(3,4)	D(4,5)	D(5,6)
−6.88	9.23	5.49	15.50	−20.64
0.009999	0.009999	0.009999	0.009999	0.009999

**TABLE 5:** Tail and quantile estimation for the generalised extreme value distribution for annual maxima with *r* = 4.

Quantiles	Temperatures (°C) (*x*_p_)	Observed number of exceedances	GEV distribution no. of exceedances
90th	32.0	4	2
95th	32.1	2	1
97.5th	32.2	1	0
99th	32.3	0	0

GEV, generalised extreme value.

The number of observations that are above the estimated tail quantile *x*_0.1_ = 32.0 are then counted and found to be 2. For the observed number of exceedances, we get 0.1 × 44 = 4.4 ≈ 4, where 44 is the number of observations available in 11 years by four order statistics.

Table 5 presents a summary of the estimated tail quantiles at different tail probabilities. The tail quantiles (temperatures) are given in column 2. The observed number of temperatures that is larger than the estimated tail quantiles is shown in column 3, while column 4 shows the corresponding number estimated using the GEV distribution. The estimation of the tail quantiles in this article establishes maximum temperatures that are expected to be exceeded. Considering the 90th quantile, for example, *x_p_* = 32 °C, is the maximum temperature that is expected to be exceeded on average once every 10 years. This is the temperature above which we expect the occurrence of extreme heat that may lead to natural hazards such as hot spells or heat waves. From the viewpoint of energy demand forecasting, this is the maximum temperature above which the demand of electricity does not increase significantly.

## Conclusion

Natural hazards are established in the literature as major causes of various massive and destructive problems worldwide. The occurrence of extremely high temperatures, heat waves and hot spells affects millions of people through several impacts. These include cases of hospitalisation, loss of lives and economic challenges. It is important that countries should always be prepared for natural hazards that cause actual disasters as uncertain events. One of the steps towards the preparation is to manage the risk of the occurrence of heat waves, which is performed in this study through modelling the frequency of the occurrence of extremely high temperatures. A brief discussion of actual disasters together with their corresponding impacts is given in this study. The main focus is on the reduction of disaster risk that could occur as a result of extreme heat. This study has demonstrated the modelling approach that is relevant for guarding against the disaster risk that could occur as a result of extreme high temperatures. The GEV_r_ distribution for *r* largest order statistics is fitted towards modelling AMDT in South Africa over the period 2000–2010. Of the 10 order statistics, *r* = 4 gives a reasonably good fit of the GEV_r_ distribution to the data. The choice of *r* = 4 as the best model is based on the deviance statistic with the support of the diagnostic tools, which are the Q–Q plots and the EDT. The asymptotic behaviour of the *r* largest order statistics model reveals the Weibull family as an appropriate distribution that can be used for modelling AMDT in South Africa. Inference is performed by analysing several return levels corresponding to the return periods as well as plotting the profile log-likelihoods for the parameters of the best model.

## Recommendations

The methodology and results of this study are important to disaster risk managers as the modelling framework demonstrated in this article is valid for modelling occurrence of extreme heat. This may be useful for guarding against risk of disasters that may occur as a result of hot spells and heat waves. The use of statistical approaches that concentrate on the mean instead of the tails of the distributions is not recommendable for modelling the occurrence of extreme temperatures. The Weibull family of distributions is recommended for modelling AMDTs in South Africa. This study is also important for power utility companies such as Eskom, South Africa’s power utility company, as demand for electricity significantly increases during a heat wave period. This will therefore help system operators to determine the amount of electricity that is consumed during a heat wave period, which will then help them to schedule and dispatch the increased demand in electricity. Extreme high temperatures cause transmission lines to sag. Combination of extreme heat and the added demand for electricity to run air conditioning causes transmission line temperatures to rise.

The use of extreme value analysis techniques such as the GEV distribution for *r* largest order statistics and the POT approaches is recommended for application in meteorology and energy demand forecasting. Areas for future research include the use of multivariate non-stationary EVT models with the parameters that are estimated based on the Bayesian estimation framework including an analysis of the duration a hot spell would last if it occurs in future, the area it will cover and how it will vary with climate and impact on mortality.

## Figures and Tables

**FIGURE 1-A1:**
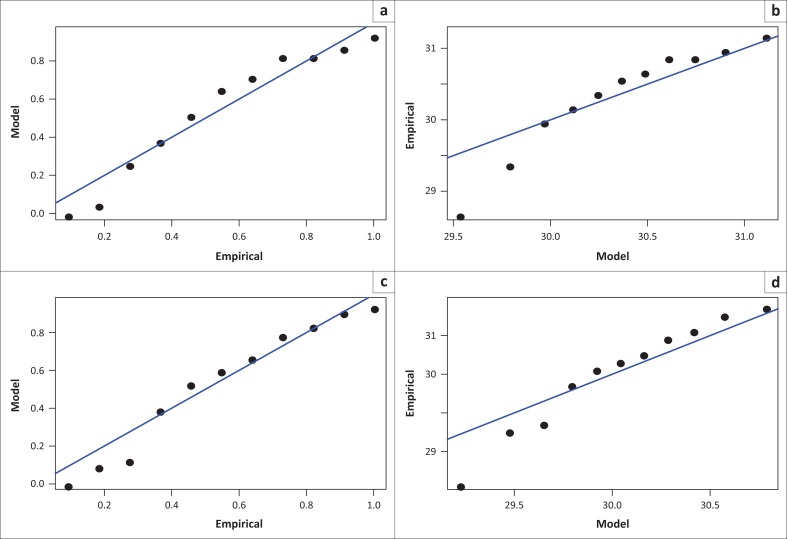
Diagnostic plots illustrating the fit of the data (annual average maximum temperature) to the generalised extreme value distribution for *r* largest order statistics model with *k* = 3 and *k* = 4, (a) probability–probability plot for *k* = 3, (b) quantile–quantile plot for *k* = 3, (c) probability–probability plot for *k* = 4, and (d) quantile–quantile plot for *k* = 4.

**FIGURE 2-A1:**
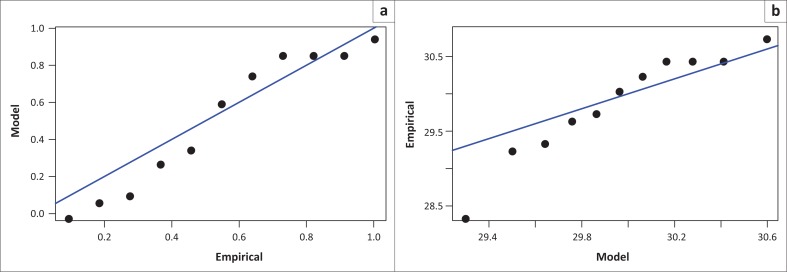

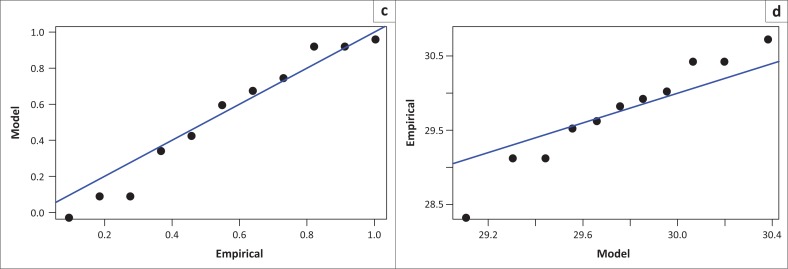
Diagnostic plots illustrating the fit of the data (annual average maximum temperature) to the generalised extreme value distribution for *r* largest order statistics model with *k* = 5 and *k* = 6, (a) probability–probability plot for *k* = 5, (b) quantile–quantile plot for *k* = 5, (c) probability–probability plot for *k* = 6, and (d) quantile–quantile plot for *k* = 6.

**FIGURE 3-A1:**
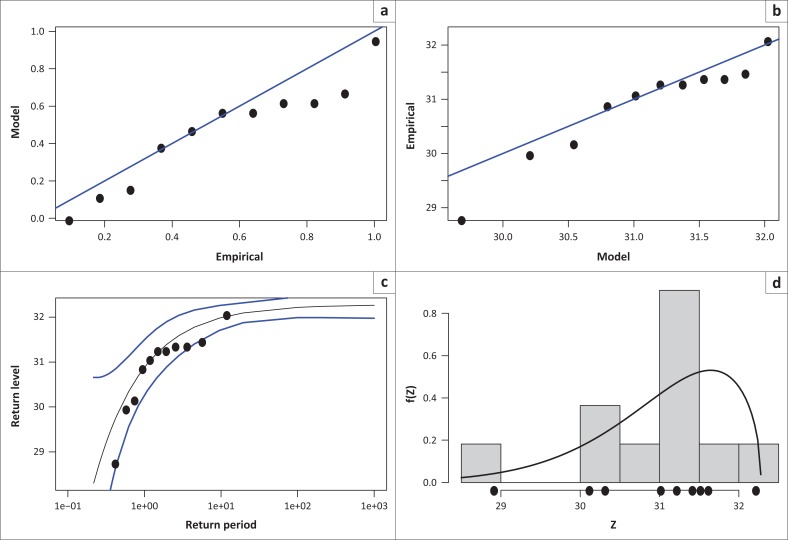
Diagnostic plots illustrating the fit of the data (annual average maximum temperature) to the generalised extreme value distribution for *r* largest order statistics model with = 4, (a) probability–probability plot for *k* = 4, (b) quantile–quantile plot for *k* = 4, (c) return level plot for *k* = 4, and (d) density plot for *k* = 4.
